# Impact of the WHO FCTC on tobacco control: perspectives from stakeholders in 12 countries

**DOI:** 10.1136/tobaccocontrol-2019-054940

**Published:** 2019-05-30

**Authors:** Lorraine Craig, Geoffrey T Fong, Janet Chung-Hall, Pekka Puska

**Affiliations:** 1 Department of Psychology, University of Waterloo, Waterloo, Ontario, Canada; 2 School of Public Health and Health Systems, Waterloo, Ontario, Canada; 3 Ontario Institute for Cancer Research, Toronto, Ontario, Canada; 4 National Institute for Health and Welfare, Helsinki, Finland

**Keywords:** global health, public policy, tobacco industry

## Abstract

**Background:**

The WHO Framework Convention on Tobacco Control (WHO FCTC), the first WHO treaty, entered into force in 2005. In April 2015, a seven-member independent expert group (EG) was established by a decision of the FCTC Conference of the Parties to assess the impact of the Treaty in its first decade.

One component of the EG’s methodology was to gather evidence on WHO FCTC impact from Parties themselves. This paper presents findings from 12 country missions on how the FCTC impacted progress on tobacco control.

**Methods:**

Between November 2015 and May 2016, EG members conducted missions in 12 countries representing each of the six WHO regions and the four World Bank economic development levels. In each country, the EG interviewed a broad range of stakeholders to assess the extent to which the FCTC had contributed to tobacco control. The primary objective was to assess whether tobacco control measures would have been developed or passed, or implemented at all, or as quickly, if there had been no FCTC. Through this counterfactual inquiry, the EG sought to determine the FCTC’s causal role.

**Conclusion:**

The FCTC was reported to have made contributions along the entire policy/regulation process: the development of a measure, building legislative and political support for a measure and its implementation. These stakeholder perspectives support the conclusion that the FCTC has played a pivotal role in accelerating and strengthening the implementation of tobacco control measures, although tobacco industry interference continues to be a significant obstacle to further advancement.

## Introduction

The WHO Framework Convention on Tobacco Control (WHO FCTC) is an international legally binding treaty that came into force in 2005, obligating governments to implement evidence-based measures to curb the tobacco epidemic. As of December 2018, the FCTC has been ratified by 181 Parties representing more than 90% of the world’s population.

There is a well-established political science literature examining factors affecting national compliance with international treaties, including empirical research to test theories and frameworks underlying compliance with environmental and human rights treaties. Studies have examined the role of international norms, domestic costs and benefits of compliance, political will, non-governmental organisations (NGOs), government administrative and technical capacity (including monetary resources and bureaucratic efficiency), and participation in treaty negotiation in decisions to ratify and implement these treaties.^1–4^

Similar frameworks and methods have been constructed to understand the implementation of the FCTC. Cairney and Mamudu examined factors influencing strong FCTC implementation based on a comprehensive review of WHO and Parties’ documents evaluating FCTC progress and interviews with more than 300 policy participants in 39 countries.^5^ Their findings point to the importance of strong health department leadership with the capacity and status within government to withstand agriculture, trade and treasury department arguments that focus on the economic value of the tobacco trade. The authors argue that many Parties struggle to implement the Treaty because a supportive policy environment is lacking. For example, China and India, home to half of the world’s tobacco users, have ratified the FCTC but are lagging behind in implementation due to challenges such as China’s state monopoly over tobacco production and India’s poor enforcement of legislation and ongoing legal challenges.^5^

This paper adds to the existing literature on global implementation of the FCTC and progress made in implementation among individual ratifying Parties by examining ways in which the Treaty influenced or did not influence progress on tobacco control in a cross-section of ratifying countries.

### Impact assessment of the WHO FCTC

At its sixth session in October 2014, the Conference of the Parties (COP6) adopted Decision FCTC/COP6(13), calling for an independent expert group (EG) to assess the impact of the FCTC on implementation of tobacco control measures and on the effectiveness of its implementation in its first decade.^6^ The Bureau of the COP selected seven independent experts from nominations sent by Parties and observers to form the impact assessment EG.

One of the primary evidence sources for the impact assessment was a qualitative process evaluation of the FCTC’s contribution to the development and implementation of tobacco control measures. The EG interviewed a broad range of stakeholders in 12 mission countries to learn about how the FCTC influenced tobacco control developments in each country.

This paper presents the main emerging themes from the 12 country missions on how the FCTC impacted progress on tobacco control. The paper highlights the ways in which the FCTC has influenced tobacco control, identifies ongoing challenges in FCTC implementation and provides insight into how progress on tobacco control might have been different without the FCTC.

## Methods

Decision FCTC/COP6(13) mandated the independent EG to conduct missions in 12 countries: three Parties selected in consultation with the Bureau, on a voluntary basis, within each of the four levels of economic development. In adherence with Decision FCTC/COP6(13), three criteria guided the selection of countries: (1) the country must be an FCTC Party, (2) the 12 Parties would consist of three Parties from each of the four World Bank economic groups, (3) reliable surveillance data should preferably be available for analysis of prevalence and (4) reliable policy evaluation data should preferably be available for analysis of strength and effectiveness of FCTC implementation.^6 7^

The selection of Parties who already had some experience with implementation of tobacco control policies was based on the rationale that conducting the study among Parties that had not yet implemented measures would have revealed little information to better understand the ways in which the Treaty impacted or did not impact policy implementation.

At its first meeting, the EG decided further that an additional criterion must be achieved: the 12 Parties should represent each of the 6 WHO regions equally. Despite the challenges of simultaneously satisfying all four requirements, a set of 12 Parties fulfilling those requirements was selected, with each of the Parties agreeing to participate in the impact assessment exercise.

The 12 country missions were conducted between 30 November 2015 and 5 May 2016. Table 1 presents the mission dates for each of the 12 selected countries. In these 12 Parties, the EG assessed the impact of the FCTC by seeking the views of relevant stakeholders, organisations and actors in tobacco control in the respective jurisdictions.^7^

**Table 1:** Mission dates and countries selected for the WHO FCTC impact assessment^7^

Mission dates	Country	World Bank category	WHO region
30 November–2 December 2015	Kenya	Low-middle	AFR
17–19 January 2016	Islamic Republic of Iran	Upper-middle	EMR
19–21 January 2016	UK	High	EUR
21–24 February 2016	Madagascar	Low	AFR
23–26 February 2016	Turkey	Upper-middle	EUR
7–10 March 2016	Sri Lanka	Lower-middle	SEAR
28–31 March 2016	Republic of Korea	High	WPR
5–8 April 2016	Uruguay	High	AMR
12–15 April 2016	Philippines	Lower-middle	WPR
18–21 April 2016	Bangladesh	Low	SEAR
25–28 April 2016	Brazil	Upper-middle	AMR
2–5 May 2016	Pakistan	Lower-middle	EMR

AFR, African Region; AMR, Region of the Americas; EMR, Eastern Mediterranean Region; EUR, European Region; FCTC, Framework Convention on Tobacco Control; SEAR, South-East Asia Region; WPR, Western Pacific Region.

Semistructured interviews were conducted by 1–3 EG members in each of the 12 mission countries, with assistance from 1 to 2 external consultants (external consultants: Paula Beltran, Daniel Ferrante, Trinette Lee and Patrick Musavuli) provided by the Convention Secretariat. Interviews were conducted with government representatives (n=217), civil society/NGO members (n=67), academics/researchers (n=25), parliamentarians (n=17), WHO country/regional representatives (n=16) and media (n=8).^7^

### Focus of the impact assessment on the possible causal role of the WHO FCTC

The focus of the EG’s inquiries was not to evaluate progress on tobacco control in each mission country, but rather to assess how the FCTC influenced or did not influence tobacco control developments. For example, did the Treaty help to strengthen laws that were passed; accelerate policy implementation; strengthen political will for measures or help to counter opposition to measures? This important distinction between assessing the strength of FCTC implementation vs the FCTC’s contributions to each country’s progress on tobacco control was incorporated into briefing materials and interview questions, and was emphasised in the EG’s communications to stakeholders at the outset of each interview session.

The EG developed an interview guide of open-ended questions for assessment of the possible causal relationship between the FCTC and policy action (see online supplementary file 1 in Fong *et al*).^7^ Interviewees were asked about the impact of the FCTC on tobacco control policies relative to a counterfactual scenario to reduce possible ‘positive response bias’ that would instead focus on a country’s tobacco control achievements and ‘negative response bias’ that criticises the FCTC and/or lack of progress in tobacco control. Counterfactual questions included, ‘Would your country have developed [tobacco control measure]/would it have been taken up in Parliament/would it have passed/would it have implemented the measure…if there was no WHO FCTC?’ Follow-up questions probed for value-added contributions of the FCTC: ‘If there was no WHO FCTC, would this same governmental action have occurred? If so, would it have happened as quickly? And would the action have been as strong?’ In all countries, the EG conveyed the distinction between needs assessment missions, which focus on tobacco control achievements as a starting point for developing methods to strengthen tobacco control efforts, and this impact assessment mission, which focused on the role of the FCTC in whatever had (or had not) been achieved in tobacco control.

10.1136/tobaccocontrol-2019-054940.supp1 Supplementary data

Data sources consisted of transcripts of over 200 hours of audio-recorded interviews conducted in the 12 countries; postmission country reports based on transcripts prepared by the ITC Project at the University of Waterloo; and postmission summaries prepared by EG members and consultants. This body of evidence was reviewed to identify content related to the impact of the FCTC and its guidelines on tobacco control.

## Results

In every mission country, stakeholders noted that the FCTC played a key role in accelerating the development and implementation of tobacco control legislation. The extent and nature of the contribution of the FCTC to tobacco control varied across the 12 countries, catalysing new policies and strategies in some countries and strengthening existing weak laws in other countries. The main impacts of the FCTC are discussed below according to six key cross-cutting themes. Supporting illustrative quotes from the country mission interviews are presented in online supplementary file 1.

### The WHO FCTC catalysed the creation of a national tobacco control law

Stakeholders in *Kenya, Bangladesh, Sri Lanka,* *Islamic Republic of* *Iran* and *Madagascar* reported that prior to FCTC ratification, there were no national tobacco control laws in place, primarily due to tobacco industry interference (TII), but after FCTC ratification, each country was able to pass a national tobacco control law.

The *Kenyan* government passed the 2007 Tobacco Control Act (TCA) 3 years after becoming an FCTC Party, overcoming long-standing barriers from TII. Interference persisted in efforts to implement the law, however with the 2010 Constitution establishing that all treaties and international agreements shall be domesticated, Kenya’s status as an FCTC Party provided a strong legal foundation for the comprehensive 2014 Tobacco Control Regulations. Multiple stakeholders in Kenya reported that strong regulations (including pictorial health warnings) would not have been advanced without FCTC ratification and its domestication under the new constitution. Even so, the Regulations were delayed until 2017 due to legal challenges.

Similarly, stakeholders in *Bangladesh* noted that although the antitobacco movement began prior to FCTC ratification in 2004, the Treaty was essential to their progress in tobacco control. During FCTC negotiations, NGOs called on the government to enact a national tobacco control law. Despite strong tobacco industry lobbying, Bangladesh passed the Smoking and Usage of Tobacco Products (Control) Act 2005, which restricted smoking in public places and tobacco advertising, promotion and sponsorship (TAPS); introduced text health warnings; and provided loans for cultivation of alternative crops. The Act was amended in 2013 to strengthen smoke-free and TAPS restrictions and to introduce pictorial health warnings on smoked and smokeless tobacco product packages. Rules to elaborate on the Amendment Act (The Smoking and Tobacco Products Usage (Control) Rule of 2015) were then issued and the 2006 Rule was repealed. However, provisions of the Act were diluted due to TII. As a tobacco growing country, Bangladesh continues to face strong tobacco industry lobbying and interference with efforts to reduce the affordability of tobacco products, strengthen smoke-free laws, implement stronger pictorial health warnings, curb point of sale advertising and reduce the consumption of smokeless tobacco.

Stakeholders in *Sri Lanka* also expressed the importance of the FCTC and its guidelines, particularly the time-bound provisions for implementation, in providing direction and guidance to accelerate tobacco control. Efforts to develop tobacco control policies were initiated in the 1990s, but there was no national comprehensive tobacco control law in Sri Lanka prior to becoming an FCTC Party in 2005. Tobacco control legislation was drafted in 1999, but due to TII it was not passed until 2006 as the National Authority on Tobacco and Alcohol (NATA) Act, No. 27. The FCTC empowered and mobilised communities and politicians, guided the content and accelerated the enactment of the 2006 NATA, and provided the legal basis for smoke-free legislation, tobacco advertising bans, taxation policies and measures to limit youth access to tobacco products. The Treaty continues to guide amendments to strengthen NATA and other related legislation. Advocacy work to implement NATA, particularly WHO FCTC Articles 8, 11, 12 and 13, were said to have contributed to tobacco denormalisation and shifts in political and social attitudes. Stakeholders noted that Article 8 guidelines, particularly statements highlighting that there is no safe level of exposure to tobacco smoke, were key to overturning the tobacco industry challenge to the smoking ban in enclosed public places in 2006.

After three unsuccessful attempts to introduce a comprehensive tobacco control law in *Islamic Republic of* *Iran* prior to FCTC ratification in 2005, the National TCA was passed in 2006, and the Executive Bylaw of National TCA in 2007. This included a comprehensive smoke-free law with strong enforcement measures and a ban on all forms of TAPS making Islamic Republic of Iran the first country in the Eastern Mediterranean Region to implement a comprehensive TAPS ban. This led to ongoing momentum to strengthen tobacco control in the country. FCTC ratification was noted as having a crucial impact on raising political support for tobacco control. By 2008, Islamic Republic of Iran had attained WHO’s highest level of implementation of FCTC Articles related to smoke-free (Article 8), health warnings and education campaigns (Articles 11 and 12), advertising and promotion bans (Article 13), cessation (Article 14), and monitoring tobacco use and prevention policies (Article 20).

Stakeholders in *Madagascar* were unanimous in expressing that Interministerial Orders and Decrees for the development and implementation of pictorial health warnings and a comprehensive smoke-free law would not have been introduced in the absence of the FCTC and its guidelines. The FCTC also guided strong taxation policies (one of the highest tax rates in Africa) and provided justification to curb tobacco industry opposition to tax increases. At the time of the interviews, a new comprehensive national tobacco control law was under development which included measures to address TII, as recommended in Article 5.3.

### The WHO FCTC strengthened existing tobacco control policies

In countries where tobacco control policies had existed prior to ratifying the FCTC, stakeholders described the important role of the Convention in strengthening policies (eg, smoke-free and pictorial warnings) and adopting new policies (eg, taxation and plain packaging) and programmes.

Stakeholders in the UK, which ratified the FCTC in 2005, noted that the process to introduce smoke-free laws was initiated before FCTC ratification. However, in Scotland, more recent measures to ban smoking in private vehicles and campaigns to reduce smoking at home were justified by continuous reference to Article 8 guidelines for implementation. The key elements of the UK’s tobacco control strategy are firmly grounded in the FCTC, and described as mutually reinforcing resulting in smoking prevalence reductions. Health policy officials contrasted policy-making on tobacco vs alcohol, noting that the FCTC enables policy proposals to be positioned as the UK’s commitment to a global tobacco control treaty which is a major advantage in building cross-party support for new health measures. The definitions and descriptions of plain packaging in Articles 11 and 13 guidelines for implementation were important references in the development of the UK’s standardised packaging policy and influential in government testimony against the legal challenge filed by four major multinational tobacco companies, which was ultimately overturned by the High Court in 2016. In addition, the 2014 EU Tobacco Products Directive is framed around meeting the European Union’s obligations under the FCTC, including requirements for pictorial health warnings in line with Article 11 recommendations and a minimum pack size of 20 cigarettes in line with Article 16. Stakeholders commented that Parties that have made the most progress in advancing domestic decision-making against tobacco are those where policy-makers have recognised the power and potential of the Treaty.

Before becoming a Party to the FCTC in 2005, *Pakistan* had introduced several basic tobacco control measures, including text health warnings, restrictions on smoking in public places and a ban on sales to minors in 2002. These measures were driven and reinforced by the country’s early ongoing involvement in the FCTC negotiation process. Stakeholders indicated that the FCTC was influential in moving tobacco control to the top of the health agenda and provided direction to strengthen existing laws. The smoke-free law was expanded in 2009 and sub-national jurisdictions (eg, Islamabad, Lahore, Karachi) enacted laws that were more stringent than the national law. In 2010, bans on youth-oriented marketing and packs containing fewer than 20 cigarettes were implemented and pictorial warnings were introduced on cigarette packages. In 2015, the size of the warnings was increased from 40% to 85% of both sides of the pack, but was not implemented due to a tobacco industry legal challenge. After an interministerial review, a phased increase was adopted—to 50% in June 2018; and 60% in June 2019.

Stakeholders in the *Philippines* explained that prior to ratifying the FCTC in 2005, a national Tobacco Regulatory Act was adopted in 2003; however, the law was weak due to the influence of the tobacco industry. The FCTC had a significant impact on advocacy to strengthen the national law resulting in stronger tobacco advertising bans in 2007 and 2008, pictorial health warnings in 2014 and tobacco tax increases between 2014 and 2016 following the introduction of the 2012 Sin Tax Law.

### The WHO FCTC mobilised and strengthened collaboration between health and non-health sectors and engagement with civil society organisations

The majority of countries described the significant impact of the FCTC in strengthening multisectoral collaboration and the participation of civil society on tobacco control. Several countries pointed out that after the FCTC was ratified, tobacco control was broadened from solely a health issue to a whole-of-government issue. Several countries reported that the Treaty’s recognition of the essential role of civil society in tobacco control has led to substantial progress on tobacco control—progress that would have been slower without the strengthened presence of civil society.

In *Kenya, Brazil* ** and *Pakistan*, mobilisation of intergovernmental policy processes and engagement of civil society were strengthened during their participation in the Treaty negotiations, resulting in strong tobacco control policies after the Convention was ratified. *Kenya* noted that the FCTC encouraged a highly active civil society that has played a vital role in supporting government tobacco control efforts and in fostering government accountability.

In *Brazil*, multisectoral collaboration on tobacco control began during the FCTC ratification process when the Ministries of Justice, Labor, and Agriculture joined with the Ministry of Health to form a national committee to negotiate this process. After FCTC ratification in 2005, collaboration across various ministries was strengthened through the formation of Comissão Nacional para a Implementação da Convenção-Quadro (CONICQ)—the National Commission responsible for FCTC implementation, consistent with Article 5.2(a). CONICQ is composed of representatives of 18 Ministries and is chaired by the Minister of Health. Stakeholders in Brazil recognised the important role of civil society since 2003 in ensuring FCTC ratification and their ongoing dedication after ratification to building support for strong implementation through research, training, media campaigns and advocacy.

In the *UK*, civil society engagement in tobacco control and overall accountability have increased since FCTC ratification. Policy-makers noted the important contribution of civil society organisations in the development and implementation of tobacco control plans and providing support to governments in responding to tobacco industry challenges. The establishment of the Framework Convention Alliance was described as having a massive impact on enhancing the effectiveness of civil society in advancing tobacco control by providing a global forum for discussion and interpretation of the FCTC.

In *Madagasca* *r*, the engagement of civil society and cross-sectoral participation was strengthened after ratification of the FCTC through the establishment of an interministerial committee on tobacco control lead by the National Office for Tobacco Control (OFNALAT).

Several countries (the *UK, Brazil, Pakistan, Madagascar* and *Republic of Korea*) discussed the importance of Article 6 and its guidelines for implementation in raising awareness across sectors of the potential for taxes to reduce tobacco affordability, generate revenue and reduce smoking prevalence. The Treaty provided the framework and rationale for ministries of health to engage with ministries of revenue and finance to increase tax and price to reduce the affordability of tobacco products. *Brazil* and the *UK* mentioned the role of the FCTC in facilitating dialogues between ministries of health and revenue leading to stronger price and tax measures. In 2011, Brazil adopted regulations to increase taxation and reduce affordability of tobacco products. Stakeholders in the *UK* noted that after ratification of the FCTC, responsibility for tobacco control shifted from solely the health sector to include other government departments and collaboration between multiple sectors was reinforced. The country’s obligations to Article 6 opened doors for discussion of taxation between health and revenue ministries which was deemed important for forthcoming discussions to reduce the tax differential between cigarettes and hand-rolled tobacco. It was also noted that the FCTC led to increased awareness among customs officials that illicit trade is a health problem and not just a revenue problem.

In *Pakistan*, stakeholders noted that the FCTC has supported the government in strengthening price and tax measures and building capacity within the Federal Board of Revenue regarding taxation of tobacco products.

*Turkey* implemented regular tax increases immediately after ratification of the FCTC. Article 6 provided the foundation for price and tax measures that contributed to lower cigarette affordability and smoking prevalence reductions.

The *Republic of Korea* asserted that while the FCTC and its implementation guidelines have not solely led to tobacco control advancements over the past decade, they have clearly played an important role, and continue to provide justification for stronger policies in the face of industry opposition. Following ratification of the FCTC in 2005, the Convention served as ‘a compass’, according to a government official. The FCTC has played a key role in strengthening cessation services, the smoke-free law, the tobacco tax increase in 2015 and the implementation of pictorial warnings in 2016. Progressively stronger action on tobacco control was influenced by changes in political leadership, the international standards set out in the FCTC and NGO-led campaigns to denormalise tobacco. The Republic of Korea’s leadership in the governing body of the Convention as host of COP5 in 2012 and holding the presidency of COP6 in 2014 were also major drivers for accelerated action.

The establishment of a strong national tobacco cessation infrastructure was viewed as one of the country’s major achievements following FCTC ratification. The Republic of Korea is one of the few countries in the world to provide nationwide government-supported smoking cessation clinics. Article 14 guidelines for implementation are used as a reference to secure financial resources to expand and increase access to affordable quality cessation services. Government officials highlighted the importance of the time-bound provisions of Articles 8 and 11 in mobilising political, public and media support for stronger policies. The FCTC has increased awareness of the public health benefits of raising tobacco taxes. Increases in cigarette prices in 2015 provided financial support for cessation services and anti-tobacco media campaigns. The FCTC serves as the overall guiding framework for the National Health Promotion Plan 2011–2020 tobacco control strategy. A common theme underlying progress on tobacco control is ‘Republic of Korea must meet international standards’. The Party actively consults the FCTC guidelines and is working towards the goal of achieving a comprehensive TAPS ban in spite of strong industry opposition.

In *Sri Lanka* and *Islamic Republic of* *Iran*, Article 6 has helped to raise awareness that strong taxation policies are an effective strategy to reduce the prevalence of tobacco use; however, stakeholders noted that tax rates have not increased sufficiently to decrease tobacco affordability.

Stakeholders in the *Philippines* noted that the FCTC contributed to the adoption of the Sin Tax Reform Act (2013) by providing the Department of Finance with key health arguments to support increasing tobacco taxes. The Sin Tax Reform Act imposed different excise tax amounts for different tobacco products depending on the retail price. Incremental increases were introduced between 2014 and 2016. From 2017, a single tax rate of 30 pesos was imposed per pack, rising 4% every year thereafter. Revenues have contributed to providing universal health coverage and to support alternative livelihood programmes.

### The WHO FCTC mobilised a global tobacco control movement through international cooperation and information exchange

Stakeholders in *Brazil, the UK, Kenya, Turkey, the Philippines* ** and *Uruguay* provided examples to illustrate the implementation of Article 20 in mobilising the creation of a strong global tobacco control community and facilitating opportunities for sharing best practices in tobacco control. For some Parties, international information exchange was mobilised during their participation in the Treaty negotiations. For example, *Brazil* participated in the first FCTC negotiation meeting in Geneva in 1999, where Canada presented their forthcoming world’s first large graphic health warnings. The Brazil delegation subsequently presented a proposal to their Minister of Health to adopt pictorial warnings based on Canada’s example. In 2002, Brazil became the second country to implement graphic pictorial warnings. Thus, the FCTC contributed to stronger tobacco control policies even before it became a treaty.

*Kenya* reported that the FCTC mobilised sharing of tobacco control experiences, challenges and possible solutions at the regional level within six countries of the East African Community.

### The WHO FCTC increased awareness of TII, prompting governments to take measures to protect tobacco control against vested interests of the tobacco industry

FCTC Article 5.3 obligates Parties to protect tobacco control policies from commercial and other vested interests of the tobacco industry, in accordance with national law. Several countries indicated the importance of Article 5.3 and its guidelines for implementation in raising awareness of TII, the primary obstacle to implementing the FCTC, and in guiding strategies to curb TII, with varying levels of effectiveness.

*Kenya* has incorporated provisions for nearly all measures under Article 5.3 guidelines in their 2014 Tobacco Control Regulations, which are the most comprehensive Article 5.3 regulations in the African region. *Brazil, the UK, Turkey, Republic of Korea, Pakistan* and the *Philippines* indicated that Article 5.3 and its guidelines for implementation have influenced the development of internal guidelines for government officials’ interaction with the tobacco industry, but that stronger measures to curb TII are needed.

### The WHO FCTC provided a supporting evidence-based legal framework to overcome challenges to tobacco control measures by the tobacco industry and others

Since the FCTC’s entry into force, the tobacco industry has initiated and supported litigation challenging various tobacco control measures around the world.^8^ Stakeholders in *Brazil* noted that every legal tobacco control measure taken towards protecting the health of its population has been challenged in court.

Stakeholders in *Sri Lanka, Kenya, Uruguay, Brazil, the UK* and *Turkey* pointed to examples to illustrate the importance of the Treaty in providing a reference for governments in their successful defences against legal challenges to new legislation. For example, the obligations of the FCTC and its guidelines for implementation were specifically recognised in decisions to uphold the 80% pictorial warnings in Sri Lanka (2014 Court of Appeal decision), Kenya’s comprehensive 2014 Tobacco Control Regulations (2016 High Court decision, 2017 Court of Appeal decision), pictorial health warnings and single presentation policy in Uruguay (2016 arbitral tribunal decision), pictorial warnings in Brazil (2009), smoke-free prisons (2015 High Court decision) and plain packaging in the UK (2016 High Court decision), and the smoke-free law in Turkey (2010 Constitutional Court decision).

In *Turkey*, a national tobacco law was introduced in 1996, but it was not compliant with the FCTC. Stakeholders indicated that after FCTC ratification in 2004, it increasingly recognised the power of the Treaty as a legal instrument to accelerate the national and international tobacco control agenda. The NGO community, with support from strong government leadership, was integral to catalysing the first national law and strong implementation of the FCTC. Reference to the FCTC and its guidelines was instrumental in parliament and in the drafting of legislation and development of action plans, as well as in the defence against the tobacco industry legal challenge to new legislation. In 2013, Turkey became the first country to attain the highest level of achievement for all of the WHO FCTC’s MPOWER measures (referring to the WHO FCTC policy package M [Monitoring], P [Smoke-Free Policies], O [Cessation], W [Warnings]: Health Warnings and Mass Media, E [Advertising Bans] and R [Taxation]). The FCTC has encouraged Turkey to implement progressive policies, such as the 2012 ban on the use of 43 additives in tobacco products, the introduction of a digital tax stamp system to reduce illicit trade in 2007, and plans to introduce plain packaging as part of the 2015–18 National Tobacco Control Program. However, stakeholders indicated that although cigarette consumption decreased between 2008 and 2012 in response to tobacco control policies, consumption has increased since 2012. They also noted challenges with non-compliance with smoke-free laws, particularly in bars.

In *Uruguay*, strong political commitment to tobacco control by President Dr. Tabaré Vásquez, coupled with the power of the Treaty as a legal instrument, led to Uruguay becoming a world leader in tobacco control. References to the FCTC are incorporated into the preamble of legislation that govern smoke-free environments; TAPS bans; and tobacco product packaging and labelling.

After *Uruguay* ratified the FCTC in 2004, it strengthened existing smoke-free legislation to become the first Latin American country, the first in the Americas region, and the first middle-income country worldwide to adopt comprehensive smoke-free legislation. The FCTC was the trigger for the 2006 comprehensive smoke-free law, which became the cornerstone for precedent-setting legislation, including pictorial warnings on 80% of the front and back of cigarette packs in 2009—the largest warnings in the world at the time, and legislation restricting each cigarette brand to a single variant in order to avoid misleading consumers about the relative safety of tobacco products. The FCTC and its implementation provided legal and evidential support that were key to defeating the tobacco industry’s legal challenge against the larger warnings and the single brand presentation policy. *Uruguay* has progressively strengthened laws to restrict youth access to tobacco, ban TAPS and to offer universal cessation treatment and other cessation support.

## Discussion

The 12 country missions conducted by the Impact Assessment EG provided clear evidence that the FCTC had strong impact in accelerating the elaboration and strengthening the implementation of tobacco control measures among Parties across a range of WHO regions and World Bank income groups. The FCTC has broadened political support for tobacco control and provided a comprehensive roadmap of legal obligations used by governments and courts to overcome TII with the introduction of new policies. By urging cross-sectoral collaboration and promoting a strong role for civil society, the FCTC has established the underlying mechanisms to support comprehensive tobacco control policies. After its first 10 years of operation, the Treaty and its guidelines continue to assist Parties at various stages of FCTC implementation in adopting new and stronger policies.

TII continues to be a major obstacle to progress on global tobacco control. While Article 5.3 of the Convention has raised awareness of tobacco industry practices and has prompted governments to develop codes of conduct and policy measures in several countries, it was clearly identified that stronger measures are needed to counter the commercial and other vested interests of the tobacco industry.

It is important to note that some countries expressed other tobacco control challenges, such as slow progress in reducing the affordability of tobacco products, lack of funding for enforcement activities and cessation services, illicit trade and lack of effective measures to support tobacco farmers in switching to alternative livelihoods. These findings are consistent with preliminary data presented in the EG’s final report from the Sixth Global Progress Report on Implementation of the FCTC, showing that in 2016, less than half of Parties had implemented six substantive articles of the FCTC, including Article 13, which requires Parties to undertake a comprehensive ban on TAPS within 5 years.^9 10^ Also, a global evidence review on the impact of the FCTC on tobacco control conducted by the International Tobacco Control Policy Evaluation Project (the ITC Project) for the EG found slow progress overall in implementing policies to regulate the contents of tobacco products (Article 9); economically viable alternatives (Article 17); protection of the environment and health of persons (Article 18); liability (Article 19); and international cooperation (Article 22).^11^ These findings are also highlighted in the EG Report.

### Limitations

The limitations of the scope of the country mission exercise mandated by the COP as a tool to assess the impact of the FCTC are recognised. The criteria for selection of the 12 mission countries (voluntary participation, available data on policy impact) precluded the participation of Parties where there has been limited or weak implementation of tobacco control policies. However, the purpose of the country missions was to examine the role and contribution of the FCTC in policy formulation and implementation in countries where tobacco control policies had been implemented and where there was at least some evaluation data available. As described in the Methods, the interview protocol, with consistent emphasis on the counterfactual questions to assess whether progress would have been achieved without the FCTC, allowed for a separation between tobacco control achievements in a country (which was not an objective of the EG) and whether the FCTC had been one of the causal factors in those achievements (which was the primary objective of the EG).

Further, the EG’s analysis of multiple sources of evidence in addition to the country missions, including the global evidence review of implementation of 17 FCTC articles,^11^ commissioned papers on the use of the FCTC in legislation and legal defences,^8^ as well as industry interference,^12^ analysis of FCTC impact on smoking prevalence,^13^ and preliminary findings from the 2016 Global Progress report^14^ gave the EG confidence that the assessment was comprehensive, transparent and thorough.

The EG in their final report to COP7 recognised that strong, coordinated and transparent application of Article 5.3 across all levels of government is the highest priority for progress in FCTC comprehensive implementation.^10^ The country mission findings are highly consistent with an analysis of national tobacco control legislation in 75 countries and the European Union, which documented extensive references to the FCTC in legislative objectives, definitions and/or substantive provisions of their tobacco control legislation or policy.^8^ Several themes consistent with the political science literature on factors influencing compliance with international treaties (human rights, environmental treaties and the FCTC) emerged from the country mission interviews, including the importance of involvement in treaty negotiation, political will, NGO participation and strong health department leadership in facilitating multisectoral collaboration.

It is encouraging that a new global action plan to promote implementation of the FCTC, the Global Strategy to Accelerate Tobacco Control (2019–2025), as well as new strategies to prevent TII were adopted at the eighth session of the Conference of the Parties in October 2018.^15 16^ These developments will be important to increasing the implementation of measures consistent with FCTC at the highest level, which has been shown to significantly prevent and decrease tobacco use.^13^

In conclusion, in its first decade, the FCTC has had significant impacts on tobacco control according to stakeholders in each of the 12 mission countries. Stakeholders were unanimous in the view that without the Treaty, tobacco control would not have advanced to the extent it had at the time of the interviews. The FCTC has elevated tobacco control as a public health priority in the national and international agendas, and provided a best practice roadmap and mechanisms to support evidence-based action on tobacco within a supporting legally binding framework. Further efforts to assist countries in overcoming TII and other obstacles to their tobacco control policies in the coming years will be important to facilitate FCTC implementation at the highest levels.

**What this paper adds**

- This paper is the first integrative summary of findings from the WHO Framework Convention on Tobacco Control (FCTC) Impact Assessment country missions.
- It provides evidence that the WHO FCTC has supported countries across all stages of policy implementation, ranging from the introduction of basic demand management policies to overcoming tobacco industry challenges against new and innovative policies.
- It illustrates that the Convention has played a key role in raising awareness of tobacco industry interference and initiating policies to curb it. However, countries need additional support to overcome tobacco industry interference and other implementation challenges.
