# Supplementary material for: Impact of the WHO FCTC on tobacco control: perspectives from stakeholders in 12 countries
Source: Tob Control. 2019 May 30;28(Suppl 2):s129–35. doi: 10.1136/tobaccocontrol-2019-054940 (PMC6589457; doi:10.1136/tobaccocontrol-2019-054940)
Supplement: Supplementary data [file tobaccocontrol-2019-054940supp001.docx]

**Supplementary File 1: Illustrative quotes from stakeholder interviews conducted in**

**the 12 mission countries**

**The WHO FCTC catalyzed the creation of a national tobacco control law**

*“If you look at the history of Kenya and legislation, many things were not moving. We signed and ratified the FCTC — it fast tracked the national process of realizing the national laws.” (Kenya)*

*“If there was no FCTC, I think probably our law would not have passed. We would still have the industry as a very strong arm in delaying and watering down the legislation. And after we passed the FCTC, we signed the FCTC, we used the FCTC to strengthen our legislation.” (Kenya)*

*“Even before the FCTC, the anti-tobacco movement in Bangladesh started to take place. But there was no legislation, there was no law.” (Bangladesh)*

*“When the government came to know that the law from 2005 was not in full compliance with the FCTC requirements, a committee comprising 7 or 8 members was formed and they sat for many meetings and they were also consulting all the FCTC articles — how to comply and how to fit those articles into new law. For example, raising high taxes and the pictorial warnings — these were not in the former law. A lot of issues were put into the amended law in 2013. It was the FCTC that was the guiding principle for the amendment, that is sure. So all the time FCTC has been working as a cornerstone for the government for legislation and amendment.” (Bangladesh)*

*“To me, what the FCTC has done is give direction and legitimacy. And it has given us goals to work towards, different articles, they are very distinct for us to be strong to. It has accelerated some of the work we have done.” (Sri Lanka)*

*“WHO FCTC highlighted that there is no safe level of exposure to tobacco smoke and that no engineering approaches can protect against tobacco smoke. This prompted the decision to amend the NATA Act to ensure a comprehensive ban and create a 100% smoke-free environment.” (Sri Lanka)*

*“FCTC helped in a big way. We based the FCTC as our big argument and then the guidelines and when they brought up things like only up to 50% (warnings), so why are you going for 80%? We said FCTC countries still are encouraged to go for more than the FCTC.” (Sri Lanka)*

*“Before the FCTC, we tried three times and we didn’t succeed. But FCTC was very helpful to get the comprehensive law legislated by the parliament and then after approval it was signed by the President so that was a step forward to support activities at the national level. And that made the government look at tobacco as an important issue.” (Islamic Republic of Iran)*

*“Although we had considerable efforts and achievements prior to ratification of the Convention, it is obvious that ratification of the Convention has had a crucial impact on strengthening our legislation on tobacco control and in practice, enforcement of its key measures. (Islamic Republic of Iran)*

*“In the last 5 years there have been changes due to the FCTC. About three or four years ago, people could smoke in the premises of the Ministry of Finance, everywhere around. But these days, you can’t see anybody smoke at all in the premises of the Ministry of Finance. This is one of the big changes I have registered.” (Madagascar)*

*“The FCTC has been used to convince the industry to comply with this measure and so it means without the FCTC there would be no measure for pictorial warnings.” (Madagascar)*

*“Taxes have increased because of the existence of the FCTC and when tobacco industries ask for these taxes to be reduced, the FCTC is the argument that convinces these tobacco industries. And that’s why they can increase the taxes – they just choose the FCTC and they accept it.” (Madagascar)*

**The WHO FCTC strengthened existing tobacco control policies**

*“I think Article 8 has been helpful to us, maybe not in the early stages of introducing smoke-free laws because we’d started that process earlier, but in the more recent changes that we’ve made around smoking in cars and also even smoking in the home. In Scotland our current mass media campaign is on smoking at home and when you look at documentation we do refer to Article 8.” (United Kingdom)*

*“In the UK we may not have done everything in the FCTC, some of it isn’t appropriate to us, about tobacco growing for example. But all the key elements of our tobacco strategy you can trace back to the FCTC and are firmly linked in the FCTC. So whether its standardized packaging or display (bans) or (Article) 5.3, all link back to the FCTC. So I think if you can’t attribute one to the other, what you can say is that they are mutually reinforcing by the success that we’ve had in reducing prevalence.” (United Kingdom)*

*“Having this Convention allows us to demonstrate that we are moving through with a group of people who are committed to an international standard. And when you’re dealing with multinational corporations, I think you need to be able to show that you are operating in a multinational framework.” (United Kingdom)*

*“When we were describing what standardized packaging might be and what the policy might encompass, we took the definitions and descriptions from the Article 11 guidelines and explained what the policy might do. So in that stage, it was incredibly useful to have that written down.” (United Kingdom)*

*“I think the problem that we might have with the FCTC for other countries, the power of it is not recognized by policy makers and I think that countries that have used the FCTC well have had policy makers that have understood the power of it. And obviously every political system will be different, but it’s those countries that have recognized the power of using the treaty I think have been able to make the most out of it of terms of contributing to internal domestic decision making.” (United Kingdom)*

*“During that period starting from 1999, Bruntland was there at WHO, Pakistan used to visit Geneva at that time of those sessions on FCTC. Before the FCTC actually came into existence there was a lot of activities going on. Each time Pakistani delegates were there, our civil society was there, our Ministry of Health colleagues were there and part of that discussion is also reflected in the laws because the people who were behind this law, they were quite exposed to the discussions which were going on there. Although we are not having all the provisions in our law because it was promulgated before the FCTC came to be. But still many things were there.” (Pakistan)*

*“We are confident that it (the FCTC) provides the basic guidelines, basic benchmark to progress on further. You can always adopt these things to have a very effective campaign, effective legislation in place, so yes, we find it very useful. And of course, just like he said, 50% minimum* [pictorial health warning size] *are the basic benchmarks. And in Article 2, it clearly states that you are encouraged to go beyond this.” (Pakistan)*

*“I think we are fully convinced that prior to FCTC there was not even a concept of having pictorial health warning. It was for the first time FCTC came into existence in 2004 and ever since we had ratified it we were reading it more closely, the FCTC and its guidelines…” (Pakistan)*

*“Right now we have a project on TAPS legislation. So Article 13 and its guidelines, that’s the one we keep on quoting and keep on discussing. I think comprehensive guidelines are there because while drafting the law we tried to incorporate all those things.” (Pakistan)*

*“The FCTC was actually pre-empted by our national law. Wherein the national law was already watered down by the industry and it came ahead of the FCTC. So on that note, what we are doing now is since we have FCTC and our national law is not as good as what we would have wanted during that time. So now we are working on having better laws that is patterned after the FCTC. We have like a national law that has smoking provisions for smoke free, we have an advertising ban, but not comprehensive and we have a health warning, but text only. But actually now that has been amended already into graphic health warnings.” (Philippines)*

*“Some of the notable achievements in the Philippines in the field of tobacco control, the most outstanding achievement I would say is the sin tax, which is Article 6. It took us 5-10 years to have the sin tax reform law passed in the senate and congress and then we also achieved a lot in terms of government policy on Article 5.3 – preventing tobacco industry interference in public health policies. And also I think we consider this an achievement even though the size is still minimal, the graphical warning law in the Philippines, which was passed 2 years ago.” (Philippines)*

**The WHO FCTC mobilized and strengthened collaboration between health and non-health sectors and engagement with civil society organizations**

*“So we have the civil society made up of groups representing women, youth, religious organizations, lawyers and I think they are very key people in this country who drove the tobacco control and they have grown to be global leaders in tobacco control. These people have really shaped the tobacco control environment in Kenya. And also provided capacity building and guidelines. And usually they are available at all times when you ask them they are really willing and ready to help at all times.” (Kenya)*

*“The FCTC encourages participation of civil society and we actually implemented that and we see the need for that so learning from example. They are also helping us to monitor the industry, article 5.3, and they also do shadow reporting.” (Kenya)*

*“FCTC has really been a milestone in enhancing tobacco control in Brazil, enhancing the health sector power to mobilize all other sectors to accomplish or to commit with FCTC goals and principles and objectives.” (Brazil)*

*“I think that the FCTC is a support for the civil society advocacy together with the legislators and I think that if we don’t have one kind of thing like that, if we don’t have a Framework Convention, if we don’t have a global movement, I don’t know if we’d have the same impact that we’ve had.” (Brazil)*

*“The FCTC creates a safe space – a climate in which tobacco control policy can flourish and creates an opportunity for civil society and for governmental organizations to pursue objectives in this area in a way that is less hindered by the huge power that is brought by the tobacco industry it allows a more level playing field.” (United Kingdom)*

*“The references to civil society and Article 5.3 in the treaty are so important in giving us a recognized place at the table in the development and implementation of the FCTC both internationally in the negotiations in the sessions of the COP but also nationally within our own parties.”(United Kingdom)*

*“Civil society getting together and having a chance to meet and discuss and engage was important then and continues to be important today and the Framework Convention Alliance is incredibly important. We have fora for discussing what the FCTC means and how we can interpret it and how we can take it going forward.” (United Kingdom)*

*“Thanks to the FCTC there is intersectoral collaboration, different ministries are already involved in tobacco control but they will try to extend this committee to all the ministries that are here in Madagascar.” (Madagascar)*

*“Since 1997, the NGO has worked on tobacco control. At that time there was no FCTC but they were convinced that tobacco control was one of their missions. The FCTC is helping them organize the fight against tobacco. They are protected by the FCTC because it’s an international law, they can use it.” (Madagascar)*

*“[before the FCTC] we didn’t have any possibility of dialogue with the tax authorities, the Minister of Finances because in their mind, the only reason for tax, tobacco taxation, was to have revenues. So it was very difficult to have any kind of dialogue. (Brazil)*

*“In 2011, when the Minister of Finance launched the decision to increase taxes, the rationale that they formally presented was Article 6 of the FCTC. It’s there, it’s a document. It was a big key milestone in the engagement of FCTC.” (Brazil)*

*The case of taxation in Brazil was one of these measures that go forward because of the FCTC, because of the role of CONICQ and integration with the other Ministries because I think it if we didn`t have this legal support, we couldn’t reach the same results.” (Brazil)*

*“The FCTC will help in the negotiations of the tax directive because we can point to the Article 6 guidelines to say – you need to do something about the very disparate tax legislation on roll your own or fine cut tobacco and manufactured cigarettes which are a key problem.” (United Kingdom)*

*“One of the things that I would point to is the opportunity given us to have a joined up approach across government. So we used the FCTC when we were talking to our colleagues in treasury, customs and revenue departments, enforcement arms and local authorities and it’s the one thing that unifies us.” (United Kingdom)*

*“What the FCTC has done is that it created the reason for the departments of revenue and customs to engage in tobacco control in a more holistic way.” (United Kingdom)*

*“Our main emphasis was on the tax as a means of revenue for the government. But in the recent past with the FCTC and the inception of NATA and major concern by the health personnel of the country more has changed. Tobacco was no more just a regular product that would bring more revenue to the government. So everybody from the policy makers up to the interest groups, everybody was talking about the other (health) aspects.” (Sri Lanka)*

*“In terms of taxes, a joint activity has been conducted by the Tax Affairs organization of Iran and the Ministry of Health to change the structure of taxes on tobacco. We have already launched this activity. Technical preliminaries have been done and it is ready to be approved by governmental cabinet ministers, as well as the parliament. Guidelines for Article 6, has been positively used for preparing this bill and restructuring the new structure. It has been definitely helpful.” (Islamic Republic of Iran)*

*“Towards the latter end of the sin tax debate in congress the proponents that were advocating for the passage of the reform sin tax law started shifting the debate from the law being a revenue-generating measure into an anti-cancer bill. So they coined anti-cancer bill specifically from the finance end. Now, that may or not necessarily been driven by the FCTC, but it surely used the FCTC arguments in passing the law.” (Philippines)*

*“As far as our ministry is concerned the FCTC has had a significant impact on the legislative advocacy around the sin tax reform, particularly of course Article 6 of the Treaty. Before the FCTC, there was no solid legal basis for price and tax measures or the tobacco excise tax as a health measure to control tobacco use, so when the Philippines committed itself to the FCTC we had a solid legal basis for pushing for the sin tax reform as a health measure. This was critical because we did not only package the reform of the excise tax system for tobacco products as a revenue measure even if we’re from the department of finance. We were doing it in accordance with the objectives of the department of health, and we were pushing it as a health measure as well. And because of the FCTC, this had solid basis. We had an international convention to refer to, to support the fact that it is an evidence based health measure. And the specific guidelines on Article 6 also confirmed the needs and the structure we were pushing for.” (Philippines)*

*“The counterfactual for me would have been certainly less activity, less commitment, or less attention to the issue. It’s not just an advocacy tool, it’s also an actionable framework, it’s really something that can be implemented that can also be traced, and monitored. And that then is kind of pervasive, provides the accountability framework. I think that is an important complimentary element of advocacy that, especially when we think about moving from advocacy to action, that there is accountability and there is an actionable framework that goes along with it. So, I think it’s always difficult to say, but I think both internally for the organization, but also for the government and for the country, this has made a tremendous impact” (Philippines)*

**The WHO FCTC mobilized a global tobacco control movement through international cooperation and information exchange**

*“I think that being part of an international treaty allowed us to exchange our experience with other countries. We have been inspired by other’s experiences and we have a sense that we we’re not alone. It’s very important. We are part of a global community. We can see that being part of this treaty is also a kind of catalytic element in order to engage other people.” (Brazil)*

*“The FCTC gives countries like the UK and other world leaders, Australia and Uruguay for example, a platform on which to go out and demonstrate leadership. And without the FCTC it would be a lot harder to be able to go out and offer global leadership on tobacco control. Without the FCTC I think we probably would have just focused on supporting work within Europe, so I think it’s given us the opportunity to be more global in the work that we’ve been able to do.” (United Kingdom)*

*“I think it is really important to have friends internationally and I think what the Framework has done is allowed people to gather resources and exchange information and that’s an exchange of information in terms of successful strategies or things you could do differently/better. But it’s also a framework to realize that you’re not alone and that you are enjoying similar challenges and it is also a vehicle for exchanging information with standard, legal and political challenges that come from big tobacco. So I think from that point of view, officials find it very helpful. I think at a political level, it’s really important to have something to refer to that is above the party. At any one time, we try as hard as possible to try and get a consensus in public health.” (United Kingdom)*

*“The Philippines is now a technical assistance provider to other member states as far as Sin Tax and Article 5.3 is concerned. Those the two big areas that the Philippines is providing aid to other member states.” (Philippines)*

*“We are always going to tell our experience, but it cannot be translated to every other country, but sharing experiences, they can provide foundation for other countries to build their own road. That’s what we do based on the expertise that we have been able to build after implementing our own measures. So we do collaborate very actively with other countries through the national tobacco control program and the International Cooperation center. We are also connected globally to other international NGOs under the FCA.” (Uruguay)*

*“Before the FCTC we were talking locally to ourselves as Kenyans. Now, the debate has shifted to regional and international levels. So you can see the impact. In fact the conference we had in 2005 brought together five East African countries to discuss the FCTC.” (Kenya)*

*“We really have improved many things after we signed the FCTC… we also know that we promoted our experience to many other countries for better implementation of tobacco control activities.” (Turkey)*

**The WHO FCTC increased awareness of tobacco industry interference, prompting governments to take measures to protect tobacco control against vested interests of the tobacco industry**

*“In the last five years, the FCTC has proved much more helpful in terms of dealing with engagement with the tobacco industry and tobacco industry lobbying. I think that’s personally where the strength and the value of the treaty has been for this country in the last five maybe seven years.” (United Kingdom)*

*“The government declaration (Local Government Declaration on Tobacco Control) and Article 5.3 component of the local declaration, the fact that it comes from the FCTC and that it has very clear guidelines has been so important to local authorities taking it forward.” (United Kingdom)*

*“I’m sure you’re aware of our Joint Memorandum Circular (JMC). Before it, we had a memorandum of understanding with Philip Morris. We used the JMC to not renew the memorandum of understanding with Philip Morris. So that’s the big difference we have before the JMC and after the JMC. Before, we were not aware that it’s prohibited to have that kind of agreement with a tobacco industry player. (Philippines)*

*“We have recently developed a draft, protocol for Article 5.3, it’s in the process for getting approval from the Ministry and it contains all the information about Article 5.3 and after approval from our Ministry, we will send to all the Ministries to follow these guidelines. Once they will receive, yes we will follow-up and then we’ll ask, look this is the requirement of Article 5.3 and we need to document all these meetings.”(Pakistan)*

**The WHO FCTC provided a supporting evidence-based legal framework to overcome challenges to tobacco control measures by the tobacco industry and others**

*“Recently I’ve been signing off witnessed statements that have referenced the FCTC and its impact on policy in the UK. We were in court and just before Christmas and that was a very important element of my witness statement and I know it was on the defense of display (ban) and vending machines. So it’s a key element for us.” (United Kingdom)*

*“After the 2014 consultation, the government went ahead with standardized packaging and as you know we’ve had legal cases since then. We have some legal cases in the European courts on the Tobacco Product Directive, which have a question on standardized packaging within those proceedings. And then we’ve also got our domestic judicial reviews on standardized packaging. The FCTC has also been referenced quite heavily in those court processes too. (United Kingdom)*

*“When the law was first amended in Turkey on the basis of the international agreement which is the FCTC, we defended ourselves in the courts by saying that this is the international treaty that Turkey is a partner. Therefore all the measures and all the changes made in this regard are based on the international agreement. And since the Articles of this international Convention were considered as a main constitution of Turkey, the tobacco industry lost all of the cases against the changes in the law. Therefore in this regard we can say that the articles of FCTC are very highly welcomed and implemented and enforced at the country level.” (Turkey)*

*“The adoption of the Convention was also important because it provide the legal framework so that we could say, well it’s an obligation for us as a Party. That made us very strong when it came to making progress in the implementation of measures. Without the Convention the situation would have been very different.” (Uruguay)*

*“Having a very clear mandate from the FCTC which is binding for our state, it will establish obligations for the state and some of them had a specific term to be complied, for example, banning advertising, 5 years, health warnings, 5 years. This is a tool that provides standards and provides indicators that are very useful when it comes to implementing and taking action. It has therefore an impact not only in the civil society, but also in the parliament.” (Uruguay)*

**Implementation Challenges**

*“Yes, there is a kind of alternative product to be cultivated, and that is some crops that hold oil seeds, which in some areas it is possible to cultivate them, but what is necessary in this regard is that we have to have a plan to support those farmers who want to actually cut their relations with the tobacco company and use the alternative one, so in this regard, we need the help of the Secretariat.” (Iran)*

*“So it is in effect, Article 5.3, but we need to do more about hundred percent implementation because always tobacco industry are under the cover, they may do something that we cannot see so…we need to warn every executive development agencies about this issue.” (Iran)*

*“The situation is disappointing despite the fact that cigarette consumption has decreased, the consumption of waterpipe has increased.” (Iran)*

*“The biggest challenge we have now is the industry. When the industry starts to take you to court then other people don’t want to get involved…. So the industry tries to penetrate by using different groups.” (Kenya)*

*“So for Kenya you’ll find that most women consume chewed tobacco and they would chew mostly it used to be older persons in specific communities. But we have a big threat now because there is a surge now, new products arriving in Kenya. Shisha, a lot of young people are using it and particularly women.” (Kenya)*

*“We should strengthen the implementation of the articles and the decrees, the orders that we have promulgated, because at the beginning people always comply, but after a while it’s like just measure and people do not comply after a while with these measures, for example, for tobacco free places, they can even smoke just in front of the sign that forbids it.” (Madagascar)*

*“So I would like to start with the problem. The problem is that tobacco is a source of income in Madagascar and it’s a challenge for us and the tobacco control program.” (Madagascar)*

*Madagascar is ready to comply with the FCTC, but there are some Ministries, that might be reluctant to some measures of the FCTC.” (Madagascar)*

*“We have this tobacco board whose task is to enhance the growth of tobacco in Pakistan. They said their agenda is to look after the wellbeing of the tobacco growers.” (Pakistan)*

*“When it comes to illicit trade, Pakistan does not have the necessary resources, whether financial nor human, to counter this situation. But that does not mean that we have not talked about it.” (Pakistan)*

*“Need to do a lot of work on Article 14 that is the treatment of tobacco dependence and smoking cessation. We have this cessation initiative and there is already a prescribed smoking cessation infrastructure for the Philippines…but unfortunately even though that is within the realm of the department of health, they still lack the capacity to implement a national smoking cessation program in the country.” (Philippines)*

*“But you know, like in India, there’s a big problem, those who are tobacco growing, their whole family and life depend upon that growing unless until there is an alternate crop and they shift to some other agriculture.” (Philippines)*

*“Areca nut packets. Some of them contain tobacco, some of them don’t contain tobacco. But it’s a new product, which is coming and it has been taken up by the school children and stopped chewing gum.” (Sri Lanka)*

*“Waterpipe is a big problem. We have a comprehensive law, but in implementation we have problems. We have to combat this problem in coordination with our enforcement agencies.” (Turkey)*

*“We have nearly 25% of non-compliance in hospitality sector, but it’s coming down throughout the years. We have a problem with bars and traditional coffee houses.” (Turkey)*

*“Our prevalence rates now are so low, but we have some really disadvantaged groups, people in prison, people with mental health problems, and the poorest people in our society with the greatest inequalities who are suffering the most from the effects of smoking.” (United Kingdom)*

*“Some of the challenges in terms of tax and pricing I think most significant among these is probably downtrading. What we’ve seen over the last several years is significant growth particularly in the cheapest cigarette category.” (United Kingdom)*

*“We do not have a standardized hospital cessation program, no hospital here in Uruguay has one or maybe some has some, but the activities regarding cessation and regarding prevention itself I think there is a lot of training to be done.”(Uruguay)*

*“Taxation which is one example, since we’ve never had a very clear strategy or clear coordination from central government. I mentioned some successes and some failures, of course we do still have a lot of things to do otherwise we would not be here today and we’re trying to organize a program to support taxation on tobacco.”(Uruguay)*
